# IgG4 Immunostaining and Its Implications in Orbital Inflammatory Disease

**DOI:** 10.1371/journal.pone.0109847

**Published:** 2014-10-10

**Authors:** Amanda J. Wong, Stephen R. Planck, Dongseok Choi, Christina A. Harrington, Megan L. Troxell, Donald C. Houghton, Patrick Stauffer, David J. Wilson, Hans E. Grossniklaus, Roger A. Dailey, John D. Ng, Eric A. Steele, Gerald J. Harris, Craig Czyz, Jill A. Foster, Valerie A. White, Peter J. Dolman, Michael Kazim, Payal J. Patel, Deepak P. Edward, Hind al Katan, Hailah al Hussain, Dinesh Selva, R. Patrick Yeatts, Bobby S. Korn, Don O. Kikkawa, James T. Rosenbaum

**Affiliations:** 1 Casey Eye Institute, Oregon Health & Science University, Portland, Oregon, United States of America; 2 Department of Medicine, Oregon Health & Science University, Portland, Oregon, United States of America; 3 Devers Eye Institute, Legacy Health Systems, Portland, Oregon, United States of America; 4 Department of Public Health and Preventive Medicine, Oregon Health & Science University, Portland, Oregon, United States of America; 5 Integrated Genomics Laboratory, Oregon Health & Science University, Portland, Oregon, United States of America; 6 Department of Pathology, Oregon Health & Science University, Portland, Oregon, United States of America; 7 Department of Ophthalmology, Emory University, Atlanta, Georgia, United States of America; 8 Department of Ophthalmology, Medical College of Wisconsin, Milwaukee, Wisconsin, United States of America; 9 Division of Ophthalmology, Ohio University, Columbus, Ohio, United States of America; 10 Department of Ophthalmology, The Ohio State University, Columbus, Ohio, United States of America; 11 Department of Ophthalmology and Visual Sciences, University of British Columbia, Vancouver, BC, Canada; 12 Department of Ophthalmology, Columbia University, New York, New York, United States of America; 13 Research Department, King Khaled Eye Specialist Hospital, Riyadh, Saudi Arabia; 14 Ophthalmology Network, Royal Adelaide Hospital, Adelaide, SA, Australia; 15 Department of Ophthalmology, Wake Forrest University, Winston-Salem, North Carolina, United States of America; 16 Department of Ophthalmology, University of California San Diego, San Diego, California, United States of America; University of Birmingham, United Kingdom

## Abstract

**Objective:**

IgG4-related disease is an emerging clinical entity which frequently involves tissue within the orbit. In order to appreciate the implications of IgG4 immunostaining, we analyzed gene expression and the prevalence of IgG4- immunostaining among subjects with orbital inflammatory diseases.

**Methods:**

We organized an international consortium to collect orbital biopsies from 108 subjects including 22 with no known orbital disease, 42 with nonspecific orbital inflammatory disease (NSOI), 26 with thyroid eye disease (TED), 12 with sarcoidosis, and 6 with granulomatosis with polyangiitis (GPA). Lacrimal gland and orbital adipose tissue biopsies were immunostained for IgG4 or IgG secreting plasma cells. RNA transcripts were quantified by Affymetrix arrays.

**Results:**

None of the healthy controls or subjects with TED had substantial IgG4 staining. Among the 63 others, the prevalence of significant IgG4-immunostaining ranged from 11 to 39% depending on the definition for significant. IgG4 staining was detectable in the majority of tissues from subjects with GPA and less commonly in tissue from subjects with sarcoidosis or NSOI. The detection of IgG4+ cells correlated with inflammation in the lacrimal gland based on histology. IgG4 staining tissue expressed an increase in transcripts associated with inflammation, especially B cell-related genes. Functional annotation analysis confirmed this.

**Conclusion:**

IgG4+ plasma cells are common in orbital tissue from patients with sarcoidosis, GPA, or NSOI. Even using the low threshold of 10 IgG4+ cells/high powered field, IgG4 staining correlates with increased inflammation in the lacrimal gland based on histology and gene expression.

## Introduction

IgG4-related disease (IgG4-RD) was first described in patients with autoimmune pancreatitis who had elevated concentrations of IgG4 in serum [1]. Shortly thereafter, in 2003, extra-pancreatic lesions were described in patients with autoimmune pancreatitis, which led to the recognition of IgG4-RD as a systemic condition [2]. Since 2003, IgG4-RD has been described in a multitude of organ systems including the pancreas, biliary tree, salivary glands, kidneys, lungs, skin, prostate, and orbit [1], [3]–[7]. Across the various organ systems, IgG4-RD is known to have a similar histopathological presentation which includes a dense lymphoplasmacytic infiltrate that is rich in IgG4+ plasma cells (IgG4+PC), storiform fibrosis, and obliterative phlebitis [8].

Ophthalmic disease is a common manifestation of IgG4-RD [9]. Patients with IgG4-immunostaining, may present with painless eyelid swelling, proptosis, or diplopia [10], [11]. The lacrimal glands, the nasolacrimal duct, and the retrobulbar region may be affected [7], [11]–[13]. A consensus report recommended the term, IgG4-related dacryoadenitis for disease in the lacrimal gland and IgG4-related orbital inflammation for disease that affects adipose tissue just posterior to the ocular globe [14].

Orbital inflammatory disease can affect orbital muscle, lacrimal gland, or adipose tissue. The most common systemic disease associated with orbital inflammation is hyperthyroidism attributable to Graves disease, also known as thyroid eye disease or TED. Sarcoidosis or granulomatosis with polyangiitis (GPA, previously known as Wegener's granulomatosis) can also cause inflammation within the orbit. Many patients with orbital inflammatory disease are classified as having nonspecific orbital inflammation (NSOI, previously known as orbital pseudotumor). Little is known as to how each of these entities might be related to IgG4-RD.

The etiology of IgG4-RD remains unclear. Although the infiltration of IgG4+PC is a defining characteristic of the disease, there is no evidence that IgG4 is directly involved in the pathogenesis. In fact, some have hypothesized that IgG4, which does not fix complement, is expressed to dampen inflammation [15]. Intriguingly, an immune response to IgG4 reportedly exacerbates rheumatoid arthritis [16].

Three studies that sought to determine the prevalence of IgG4 -immunostaining among patients with orbital inflammation found very discrepant results with prevalence ranging from 4 to 52% [7], [17], [18]. In part, this relates to the definition of a positive case. Some studies have used a threshold of 10 IgG4+PC/high powered field (hpf) [9], [18]. Other studies have used thresholds of up to 30 IgG4+cells/hpf or a minimum ratio of IgG4+:IgG+PC of 0.40 or a combination thereof [11], [17]. Some have suggested that IgG4-immunostaining has immense clinical implications that frequently indicate a multisystem disease which is highly likely to respond to rituximab therapy [19], [20]. Accordingly an understanding of the prevalence of IgG4 immunostaining among patients with orbital inflammation has potential clinical and therapeutic implications. We sought to clarify the implication of IgG4 immunostaining in the orbit by studying tissue from patients with a variety of orbital inflammatory diseases. We correlated the detection of IgG4+ plasma cells in tissue with the specific diagnosis as well as with inflammation, fibrosis, and gene expression.

## Materials and Methods

### Human subjects and tissues

This study was approved by the Institutional Review Boards (IRB) at Oregon Health & Science University, Columbia University, University of California San Diego, Wake Forest University, Medical College of Wisconsin, and Mount Carmel (Ohio) and by the University of British Columbia Clinical Research Ethics Board, the Royal Adelaide Hospital Research Ethics Committee, and the King Khaled Eye Specialist Hospital Human Ethics Committee/Institutional Review Board. This study was in compliance with the Helsinki Declaration. Formalin-fixed paraffin-embedded (FFPE) samples were obtained from 9 contributing centers. Data were analyzed anonymously and written informed consent was obtained when required by the local IRB or ethics committee.

The diagnoses of nonspecific orbital inflammation, sarcoidosis, granulomatosis with polyangiitis, thyroid eye disease, and normal were based on the clinical and histopathological information obtained and submitted by orbital disease specialists and ocular pathologists from their respective institutions. Biopsies from a total of 109 subjects were used (22 controls with no known orbital disease, 42 NSOI, 6 GPA, 12 sarcoidosis, 26 TED) (Table 1). Among the 6 subjects with a diagnosis of GPA, the antineutrophil cytoplasmic antibody (ANCA) test was positive in four of five and unknown in one. Of those with a positive test, 3 had a cytoplasmic ANCA pattern. The subject with a negative ANCA had pulmonary disease in addition to the orbital disease. One other subject with GPA had renal disease, but the others had a limited form of GPA. Among the 12 subjects diagnosed with sarcoidosis, all had non-caseating granulomata present in the lacrimal or orbital adipose tissue biopsy. Adenopathy was present on chest CT scan in six. In 5 subjects, results of CT scanning and/or biopsy outside of the orbit were not known or not performed. The control tissue was obtained during surgery on eyes with non-inflamed orbits, such as blepharoplasties and enucleations. Multiple specimens were evaluated for 7 patients, three of whom had both orbit and lacrimal gland biopsies. In total there were 119 tissue biopsies (74 orbital tissue, 45 lacrimal gland) used for histopathological and immunohistochemical analysis.

**Table 1:** Case demographics.

Diagnosis	Number of subjects	Age Median (Q1–Q3)	Male:Female
Control (normal)	22	66.5 (58.9–71.0)	7∶15
TED	26	55.0 (44.6–61.0)	7∶20
GPA	6	47.1 (27.1–49.0)*	2∶04
NSOI	42	48.1 (37.6–64.3)	15∶27
Sarcoidosis	12	39.8 (31.7–49.4)*	4∶08

*Ages are not available for 4 subjects.

All samples were reviewed and scored without reference to the indications for biopsy or other clinical information. Two slides from each specimen were stained with hematoxylin and eosin for histopathological evaluation. These features were independently re-evaluated by two ocular pathologists (D.J.W. and H.E.G.). The features included degree of fibrosis, degree of inflammation, presence of obliterative phlebitis, and presence of storiform fibrosis. The degrees of fibrosis and degree of inflammation were quantified as absent (0), mild (1), moderate (2), or severe (3). For most samples, the presence or absence of lacrimal gland tissue was confirmed by the presence or absence of lacrimal gland mRNA.

### Immunohistology

Two serial sections from each specimen were used for immunohistochemical evaluation. One slide was stained for IgG4+ plasma cells (antibody from The Binding Site, San Diego, Ca, 1∶15000 dilution) and the other for IgG+ plasma cells (antibody from Dako, 1∶10,000 dilution). Immunostaining used Ventana automated instruments with Ultraview detection (Ventana, Tucson, AZ). Two surgical pathologists (M.L.T. and D.C.H.) independently counted the numbers of IgG4+ cells in three high power fields (hpf, 400X, about 0.3 mm^2^) and the IgG+ cells in the corresponding fields of the paired slides. These data are expressed as the mean number of positive cells per hpf. For the subjects for whom more than one specimen of the same tissue type was stained, the overall mean was used for the statistical analysis.

### RNA extraction and microarray

For each FFPE specimen, multiple 10 µm sections were collected and total RNA was extracted with miRNeasy FFPE kits (Qiagen, Valencia, CA) according to the manufacturer's protocol. RNA concentrations were determined by UV absorbance. RNA was prepared for microarray analysis using the SensationPlus FFPE Amplification and 3′ IVT Labeling kit (Affymetrix, Santa Clara, CA). 50 ng of RNA was used for the majority of samples with a minimum input of 20 ng RNA for samples in which RNA yields were limited. Biotin-labeled cDNA targets were hybridized with a GeneChip Human Genome U133 Plus 2.0 array (Affymetrix, Santa Clara, CA) according to standard Affymetrix protocol. This array contains over 54,000 probe sets for 47,000 transcripts and variants. Following hybridization, arrays were stained and scanned using the GeneChip Scanner 3000 7G system (Affymetrix). Image processing and initial quality control analysis were performed using Affymetrix GeneChip Command Console (AGCC) v. 3.1.1 and Affymetrix Expression Console v. 1.1 software, respectively.

### Statistical analysis

Simple statistical tests were done with chi-square tests, Mann-Whitney U-test, or Student's t-test as appropriate [21]. Affymetrix CEL files were preprocessed by the Robust Multiarray Analysis. After normalization, linear models were fitted to test potential differences in gene expression profiles due to elevated concentrations of IgG4 while controlling for diseases and batch effects. We used ‘affy’ and ‘limma’ packages of Bioconductor (http://www.bioconductor.org) in the R project for statistical computing (http://www.r-project.org).

## Results

Altogether, 119 orbital adipose tissue and lacrimal gland tissue samples from 108 subjects were evaluated (Table 1). Our control group consisted of 22 subjects without orbital disease. The disease cases included 26 with TED, 6 with GPA, 42 with NSOI, and 12 with sarcoidosis. Of the 108 subjects, about one third was male. The collective median reported age at the time of biopsy was 54.0 years (range, 12.0–96.1 years). The ages shown in Table 1 reflect the behavior of each disease. Healthy control tissue was primarily available from older subjects and this group is older than each of the four diseased groups by Mann-Whitney U test (p<0.01). However, the TED group is also a negative control and does not differ significantly in age from the other 3 diseased groups. Furthermore, we found no significant association between age and positive staining for IgG4 among the subjects with NSOI, GPA, or sarcoidosis (see below).

IgG4+PCs were rarely seen in tissues from control or TED subjects (Table 2). The frequency of IgG4+PCs varied considerably among subjects in the other disease groups as illustrated in Figure S1 in File S1; pairs of images from three subjects with NSOI show heavy, light, and no staining for IgG4 and IgG. For the five subjects for whom more than one specimen of the same tissue type was stained, the mean counts were used for statistical analysis. When classified with the low threshold of ≥10 IgG4+PC/hpf, five of six (83%) GPA cases were IgG4+. Sixteen of 42 (38%) of NSOI cases were IgG4+, and five of twelve (42%) of sarcoidosis cases were positive. Only a few cases had counts that met either of the more stringent thresholds of having at least 30 IgG4+PC/hpf or at least 40% of the plasma cells being IgG4+ (Table 2).

**Table 2:** A minority of subjects with inflamed orbits have markedly high IgG4+PC counts.

		IgG4+ PC/hpf				IgG4+ PC/hpf >30 and IgG4+PC/IgG+PC ≥0.4
		<10	10–29	30–99	≥100	
*Lacrimal Gland*						
	Control	7	-	-	-	-
	TED	4	-	-	-	-
	NSOI	15	4	2	1	1
	Sarcoidosis	3	3	1	-	-
*Orbital fat*						
	Control	15	-	-	-	-
	TED	25	-	-	-	-
	GPA	1	1	3	1	4
	NSOI	11	2	5	2	2
	Sarcoidosis	5	1	-	-	-

The number of subjects in each category is shown.

IgG4+PC infiltration was observed in both lacrimal gland and orbital adipose tissue samples (Table 2). In total, 11 of 29 (38%) lacrimal gland tissue samples from non-TED orbital inflammation patients were found to be IgG4+ based on the least stringent criterion. 15 of 35 (43%) orbital adipose tissue samples from non-TED orbital inflammation patients were found to be IgG4+. Both a lacrimal gland and an orbital adipose tissue sample were studied for one subject with sarcoidosis. Both of these samples were determined to be IgG4- and were included in Table 2.

Among the non-TED orbital inflammation cases, there was no significant correlation between IgG4+ status and gender or the mean age at biopsy based on IgG4+ immunostaining defined as at least 10 positive cells per hpf (Table 3). Examining only the NSOI samples from orbital adipose tissue, there was a trend for IgG4- samples to come from older subjects, by Mann-Whitney U test (p = 0.078, excluding one 96.1 year old outlier). There was no real significant difference in age and IgG4 status for NSOI affecting the lacrimal gland.

**Table 3:** IgG4 status is independent of age and gender.

		IgG4+	IgG4-	Sex Ratio (Male:Female)	
		Median(Q1–Q3)	Median(Q1–Q3)	IgG4+	IgG4-
*Lacrimal Gland*					
	NSOI	44.2(43.3–50.5)	45.9(37.8–61.4)	1.3∶1	1∶2.8
	Sarcoidosis	30.7(21.3–40.0	35.1(32.1–37.5)*	1∶3	1∶2
*Orbital adipose tissue*					
	GPA	48.1(41.3–50.37*	27.1	1∶1.5	0∶1
	NSOI	40.0(35.6–53.5)	69.3(44.2–72.8)	1∶2.5	1∶2.7
	Sarcoidosis	—*	53.9(45.0–58.9)	0∶1	1∶1.5


*Ages are not available for 4 subjects.

### Correlation of IgG4 staining with inflammation or fibrosis

**Figure 1:**
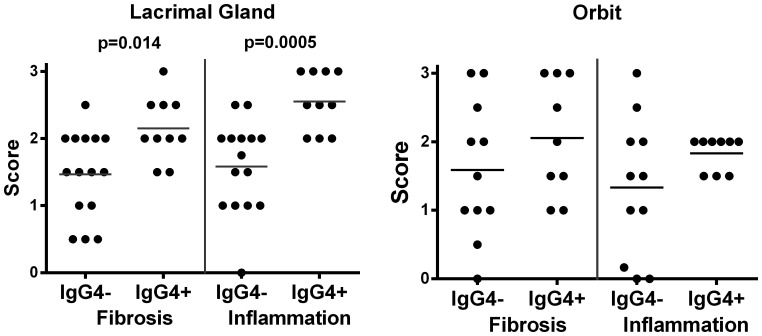
Lacrimal tissues from NSOI patients with at least 10 IgG4+ PC/hpf show increased fibrosis and inflammation. IgG4+ orbit tissues lack the lowest fibrosis and inflammation scores. Each symbol represents the score for one subject. P-values are based on Mann-Whitney U-test.

No storiform fibrosis or obliterative phlebitis was noted in any of the tissue samples. A comparison of the fibrosis and inflammation scores between IgG4+ and IgG4- cases showed a significant increase in fibrosis and inflammation in the lacrimal gland tissue among IgG4+ NSOI patients (Mann-Whitney U test, p = 0.014 and p = 0.005, respectively) (Figure 1). Although in the orbit only the IgG4- group included samples with IgG4 counts ≤0.5, these fibrosis and inflammation scores were not statistically different. For these comparisons, the correlation relied on the least stringent criteria to classify IgG4 status.

### Correlation of treatment with IgG4 staining

**Figure 2:**
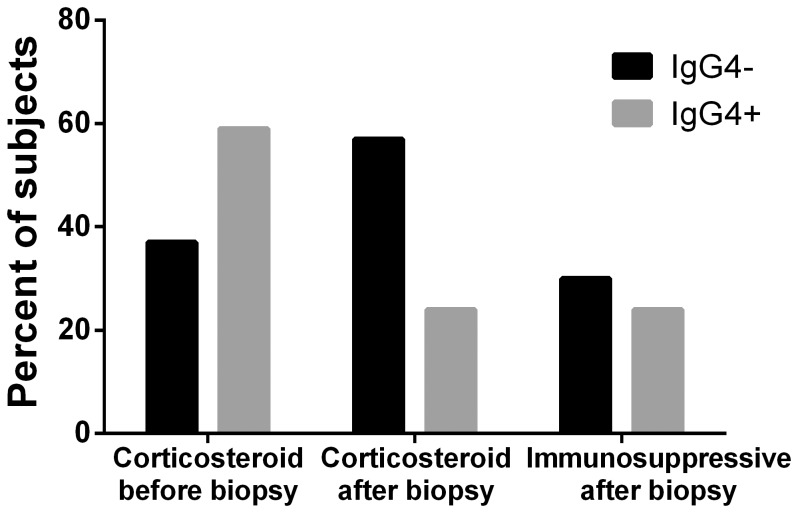
Patients with NSOI, GPA or sarcoidosis with at least 10 IgG4+ PC/hpf were not more likely to be treated with corticosteroid or other immunosuppressive therapy. These results are based on chi square testing for 30 IgG4- and 17 IgG4+ subjects for whom treatment data were available.

We employed the prescription of corticosteroid or other immunosuppressive as a surrogate index for severity, reasoning that more severe disease was more likely to prompt potentially toxic therapy. Using chi square analysis, patients with NSOI, GPA, or sarcoidosis who had at least 10 IgG4+cells/hpf were not more likely to be treated with corticosteroid (p = 0.3) and not more likely to receive additional immunosuppression after the biopsy (p = 0.7). A higher percentage of the IgG4+ patients started corticosteroid treatment prior to their biopsy, whereas the opposite was true for the IgG4- group (Figure 2). We further compared the maximum dose of prednisone given prior to biopsy to those who were IgG4 + with those who were IgG4 negative. The values (52.5±18.9 mg/day and 55.8±27.1 mg/day) respectively did not differ between the two groups. Six patients received an injection of triamcinolone as part of their therapy, and these were equally divided between the IgG4+ and IgG4- groups.

Because no subject with TED had substantial staining for IgG4, we reviewed the therapy for these subjects in case the absence of staining was a result of treatment. Of the 26 subjects with TED in this study, two were receiving prednisone at the time of biopsy, 5 had previously been treated with prednisone between 3.5 months and 3.5 years before the biopsy, 13 had not received any medications, and in 6 subjects, the prior treatment was unknown. Thus treatment is an unlikely explanation for the absence of IgG4 staining among subjects with TED.

### IgG4 staining and multisystem disease

IgG4 staining has been proposed as a marker of multisystem disease [2]. GPA by definition is a multisystem disease and sarcoidosis tends to involve multiple tissue sites. Fifteen subjects in our study had NSOI and IgG4 immunostaining. Of these 15, we were able to review the medical history of 12 in detail. None of these 12 had evidence for a multisystem disease, including the two subjects who had greater than 30 IgG4+ cells per hpf and an IgG4:IgG cell ratio of >0.4.

### IgG4 immunostaining and gene expression

We compared RNA transcripts in tissues that stained for IgG4 plasma cells with tissues from subjects with NSOI, GPA, or sarcoidosis and little or no IgG4 staining. We used a positive staining threshold of at least 10 IgG4+ cells/hpf and a threshold for gene expression change of at least 1.5-fold difference with a false discovery rate adjusted p-value of <0.05. In lacrimal gland samples, we detected 98 probes sets with increased signals and 4 that had decreased signals. For tissue from the orbital adipose tissue, 100 probe sets had increased signals and 22 were decreased. The complete lists of probe sets indicating significant expression differences are in Tables S1 and S2 in File S1.

Examples of probe sets with elevated signals in IgG4+ orbital adipose tissue (Table 4) include several immune response related genes. Of the 100 probe sets with higher signals in IgG4+ orbital adipose tissue, 16 are for light or heavy chain immunoglobulin transcripts (Table S1 in File S1). These results are consistent with the inflammation scores shown in Figure 1 in that higher scores were more frequent in the IgG4+ tissues. Table 4 also lists examples of probe sets with lower signals in the IgG4+ orbit tissues.

**Table 4:** Examples of gene expression differences comparing IgG4+ to IgG4- orbital tissue from subjects with NSOI, GPA, or sarcoidosis.

Probe Set	Gene Symbol	Fold Change	FDR P-value	Gene Title	A Gene Ontology Biological Process
***Probe sets with increased levels***					
211639_x_at	IGH; IGHA1; IGHA2; IGHD; IGHG1; IGHG3; IGHG4; IGHM; IGHV4-31	3.33	0.029	Immunoglobulin heavy locus	Immune response
216829_at	IGK; IGKC	3.06	0.013	Immunoglobulin kappa locus; immunoglobulin kappa constant	Immune response
205242_at	CXCL13	2.99	0.045	Chemokine (C-X-C motif) ligand 13	T and B cell chemotaxis
242020_s_at	ZBP1	2.80	0.048	Z-DNA binding protein 1	Positive regulation of type I interferon-mediated signaling pathway
234477_at	IGHA1; IGHV4-31	2.52	0.022	Immunoglobulin heavy constant alpha 1; immunoglobulin heavy variable 4-31	Immune response
205884_at	ITGA4	2.40	0.049	Integrin, alpha 4 (antigen CD49D, alpha 4 subunit of VLA-4 receptor)	Cell adhesion
217227_x_at	IGLV1-44	2.40	0.039	Immunoglobulin lambda variable 1-44	Immune response
216541_x_at	IGHG1; IGHM	2.18	0.045	Immunoglobulin heavy constant gamma; immunoglobulin heavy constant mu	Immune response
211648_at	IGHG1; IGHM	1.98	0.034	Immunoglobulin heavy constant gamma 1; immunoglobulin heavy constant mu	Immune response
223565_at	MZB1	2.13	0.011	Marginal zone B and B1 cell-specific protein	Positive regulation of immunoglobulin biosynthetic process
204562_at	IRF4	2.08	0.026	Interferon regulatory factor 4	interferon-gamma-mediated signaling pathway
1558561_at	HM13	2.01	0.002	Histocompatibility (minor) 13	Proteolysis
208083_s_at	ITGB6	1.96	0.043	Integrin, beta 6	Cell adhesion
201688_s_at	TPD52	1.86	0.019	Tumor protein D52	B cell differentiation
225435_at	SSR1	1.86	0.020	Signal sequence receptor, alpha	Activation of signaling protein activity involved in unfolded protein response
***Probe sets with decreased levels***					
217187_at	MUC5AC	−2.77	0.015	Mucin 5AC, oligomeric mucus/gel-forming	Extracellular fibril organization
201044_x_at 226578_s_at	DUSP1	−2.34 −1.75	0.038 0.014	Dual specificity phosphatase 1	Inactivation of MAPK activity
210226_at	NR4A1	−2.27	0.035	Nuclear receptor subfamily 4, group A, member 1	Positive regulation of endothelial cell proliferation
207008_at	CXCR2	−1.82	0.038	Chemokine (C-X-C motif) receptor 2	Dendritic cell chemotaxis
1559960_x_at	SYCE1L	−1.77	0.030	Synaptonemal complex central element protein 1-like	Meiosis
226765_at	SPTBN1	−1.75	0.047	Spectrin, beta, non-erythrocytic 1	Mitotic cytokinesis
227404_s_at	EGR1	−1.71	0.044	Early growth response 1	Negative regulation of transcription from RNA polymerase II promoter
206869_at	CHAD	−1.68	0.027	Chondroadherin	Cartilage condensation
1553572_a_at	CYGB	−1.67	0.010	Cytoglobin	Oxygen transport
208333_at	LHX5	−1.65	0.025	LIM homeobox 5	Regulation of cell proliferation
216904_at	COL6A1	−1.64	0.029	Collagen, type VI, alpha 1	Extracellular matrix disassembly
229207_x_at	RNF187	−1.62	0.017	Ring finger protein 187	Positive regulation of cell proliferation
1555380_at	ADAMTS4	−1.62	0.013	ADAM metallopeptidase with thrombospondin type 1 motif, 4	Skeletal system development
203792_x_at	PCGF2	−1.61	0.036	Polycomb group ring finger 2	Negative regulation of transcription from RNA polymerase II promoter
206128_at	ADRA2C	−1.60	0.043	Adrenoceptor alpha 2C	Activation of MAPK activity by adrenergic receptor signaling pathway

Probe sets were selected for expression increases or decreases and ample annotation. A relevant Gene Ontology Biological Process (http://geneontology.org/) is listed for each gene.

Examples of lacrimal gland transcripts with differential expression in IgG4+ tissues are listed in Table 5. Unlike orbital adipose tissue, no increased expression of immunoglobulin genes was detected. This is likely due to high levels of immunoglobulin transcripts in normal lacrimal gland that might obscure detection of additional antibody transcripts. Nonetheless, the profile of genes with increased transcript levels in the IgG4+ lacrimal gland tissues is consistent with more lymphocytic activity than in the IgG4- tissues.

**Table 5:** Examples of gene expression differences comparing IgG4+ to IgG4- lacrimal gland tissue from subjects with NSOI, GPA, or sarcoidosis.

Probe sets with increased levels					
Probe Set	Gene Symbol	FC	FDR p value	Gene Title	Gene Ontology Biological Process
228599_at	MS4A1	3.51	0.004	Membrane-spanning 4-domains, A1	B cell activation
221969_at	PAX5	3.48	0.005	Paired box 5	Regulation of transcription
217422_s_at 38521_at 204581_at	CD22	2.87 2.48 2.14	0.016 0.028 0.009	CD22 molecule	Cell adhesion
219014_at	PLAC8	2.83	0.030	Placenta-specific 8	Regulation of cell proliferation
209995_s_at	TCL1A	2.74	0.031	T-cell leukemia/lymphoma 1A	Multicellular organismal development
1558662_s_at 222915_s_at	BANK1	2.55 2.44	0.012 0.020	B-cell scaffold protein with ankyrin repeats 1	B cell activation
221601_s_at	FAIM3	2.37	0.023	Fas apoptotic inhibitory molecule 3	Regulation of apoptotic process
235400_at	FCRLA	2.37	0.017	Fc receptor-like A	Cell differentiation
1564310_a_at	PARP15	2.35	0.047	Poly (ADP-ribose) polymerase family, member 15	Regulation of transcription
205544_s_at	CR2	2.27	0.032	Complement component receptor 2	Complement receptor mediated signaling
35974_at 204674_at	LRMP	2.26 2.19	0.009 0.008	Lymphoid-restricted membrane protein	Vesicle targeting
211861_x_at	CD28	2.18	0.038	CD28 molecule	Inflammatory response to antigenic stimulus
**Probe sets with decreased levels**					
212793_at	DAAM2	−1.52	0.033	Dishevelled associated activator of morphogenesis 2	Actin cytoskeleton organization
202068_s_at	LDLR	−1.53	0.028	Low density lipoprotein receptor	Receptor-mediated endocytosis
201148_s_at	TIMP3	−1.57	0.034	TIMP metallopeptidase inhibitor 3	Negative regulation of peptidase activity
221747_at	TNS1	−1.60	0.014	Tensin 1	Fibroblast migration
36829_at	PER1	−1.61	0.009	Period circadian clock 1	Circadian regulation of gene expression
218245_at	TSKU	−1.68	0.014	Tsukushi, small leucine rich proteoglycan	Negative regulation of Wnt receptor signaling pathway

Probe sets were selected for expression increases of more than 2.1 fold or decreases of more than 1.5 fold, p values <0.5, and sample annotation. A relevant Gene Ontology Biological Process (http://geneontology.org/) is listed for each gene.

## Discussion

To our knowledge, our study is the first study to compare the prevalence of IgG4 staining among various inflammatory conditions within the orbit. A recent report on IgG4 immunostaining in skin disease similarly concluded that IgG4 staining is detectable in a variety of clinical entities [22]. Wallace and colleagues [23] described patients with IgG4 associated pachymeningitis. Their series included patients who had either meningeal sarcoidosis or GPA, some of whom stained positively for IgG4 [23]. Strehl and colleagues found positive IgG4 staining in a variety of conditions including rheumatoid synovitis and the inflammatory response around a carcinoma. [24] We believe that ours is the first study to detect IgG4 immunostaining in tissue from patients with sarcoidosis affecting the orbit. A report from Japan showed that nearly 25% of patients with lymphoproliferative disease within the orbit have IgG4+ cells in orbital tissue [25]. Another study found that 50% of subjects with xanthogranuloma in the orbit show IgG4 immunostaining [25]. Chang et al. noted the presence of IgG4+ plasma cells in GPA patient biopsies taken from various sites including the majority of periorbital biopsies [26]. Similarly we reported that renal biopsies from patients with GPA can stain positively for IgG4 [27].

Considering only the non-TED orbital inflammation cases, we found the prevalence of IgG4+ immunostaining to be 36% based on a cutoff of ≥10 IgG4+PC/hpf. However, this cutoff is controversial in the literature. In 2008, Sato et al. published one of the largest IgG4-immunostaining studies [7]. Applying a cutoff of >10 IgG4+cells/hpf, they found a prevalence of 19% among patients with ocular adnexal lymphoproliferative disorders. This paper was criticized for setting too low of a threshold for determining IgG4 status [28]. Most papers set a minimum threshold of 30 IgG4+PC/hpf [29]. Using this standard, the prevalence of IgG4-immunostaining would decrease to 15 of 64 (23%). Alternatively, some studies have used the more stringent criteria of ≥10 IgG4+PC/hpf and an IgG4+:IgG+ ratio of ≥0.4 [7], [30]. Using this stringent criterion, the prevalence of IgG4-immunostaining in our study would be 7 of 64 (11%).

Our study is the first to analyze RNA transcript expression in tissue that contains IgG4 plasma cells. We based this analysis on the least stringent definition of IgG4 staining for a practical reason; other definitions of IgG4 positivity would have provided too few samples for accurate statistical analysis. We believe that this analysis lends support for defining significant IgG4 staining within the orbit using the criterion of at least ten cells staining per hpf.

Unlike the histopathological presentation in IgG4-RD in other organ systems, we did not find storiform fibrosis or obliterative phlebitis in any of our samples, regardless of the tissue type. A consensus statement on the pathology IgG4-RD released in 2012 noted that the lacrimal gland is an exception to the general rule that storiform fibrosis should be a main criterion for diagnosis [13].

Only two of the biopsies with IgG4 staining as detected in our study were previously identified as IgG4 + by the referring center. As some consider IgG4 to be a marker of more severe disease, knowledge of IgG4 staining could have prompted more aggressive therapy which was not an issue in our study in which very few patients were known to be IgG4+. It is also possible that treatment with prednisone would diminish the number of IgG4+ cells in a biopsy. While we cannot exclude this possibility, there was no statistically significant difference between the rate of IgG4 positivity among those who took prednisone before the biopsy and those who did not take prednisone.

Most experts consider IgG4-RD a distinct clinical entity that includes the histological implication of increased fibrosis and the therapeutic implication of a likely response to rituximab therapy [12], [19], [20]. Our data could be interpreted in an alternative way: IgG4 staining identifies a subset of patients with diagnoses such as sarcoidosis, GPA, or NSOI. Those patients whose tissue stains for IgG4 have more inflammation and fibrosis, especially within the lacrimal gland. Our data cannot exclude a third possibility that both hypotheses are true: IgG4-immunostaining identifies a subset of patients with entities such as GPA, sarcoidosis, or NSOI and IgG4 staining could identify some patients with a syndrome distinct from GPA, sarcoidosis, or NSOI. Since NSOI already subsumes a presumed variety of entities, the latter hypothesis would fail to distinguish NSOI patients who are IgG4+ from the entity of IgG4-RD. We recently reported that 70% of patients with orbital inflammatory disease respond favorably to therapy with rituximab [31]. Thus therapeutic response also might not consistently distinguish the IgG4+ subset from the IgG4 – subset.

In summary, we conducted a multi-centered, international study on the prevalence of IgG4-immunostaining in orbital inflammation allowing us to compare the prevalence of IgG4+PC infiltration in different entities affecting the orbit. Unfortunately, due to the limitations of our database and incomplete clinical information, we were unable to examine the relationship between our cases and IgG4 serum concentrations. In addition, by the nature of our multi-centered approach, we were reliant on diagnoses from different institutions. In a minority of instances we lacked complete information on the therapy or diagnostic evaluation. Although the clinical implications of IgG4 staining in the orbit require further investigation, our data support the rationale to target B cells in therapy. Regardless of whether IgG4-immunostaining is considered a distinct clinical entity or a subset of other entities, subjects with an IgG4+ tissue infiltrate display an identifiable gene expression profile consistent with a heightened inflammatory response. Our study should help physicians better interpret the implications of IgG4 staining in orbital tissue.

## Supporting Information

File S1 Figure S1, Representative images illustrating the variability of IgG4 and IgG staining in orbit adipose tissue from subjects with NSOI. Table S1, Comparison of gene expression in IgG4+ and IgG4- orbit adipose tissues. Table S2, Comparison of gene expression in IgG4+ and IgG4- lacrimal gland tissues.
(DOCX)
