# Supplementary material for: IgG4 Immunostaining and Its Implications in Orbital Inflammatory Disease
Source: PLoS One. 2014 Oct 10;9(10):e109847. doi: 10.1371/journal.pone.0109847 (PMC4193851; doi:10.1371/journal.pone.0109847)
Supplement: File S1 — Figure S1, Representative images illustrating the variability of IgG4 and IgG staining in orbit adipose tissue from subjects with NSOI. Table S1, Comparison of gene expression in IgG4+ and IgG4- orbit adipose tissues. Table S2, Comparison of gene expression in IgG4+ and IgG4- lacrimal gland tissues. (DOCX) [file pone.0109847.s001.docx]

| **Figure S1.** Representative images illustrating the variability of IgG4 and IgG staining in orbit adipose tissue from subjects with NSOI. Each pair of images shows approximately the same region of tissue stained for IgG or IgG4. | | | |
| --- | --- | --- | --- |
|  | **Pair 1 – Heavy** | **Pair 2 – Light** | **Pair 3 – None** |
| **IgG** | 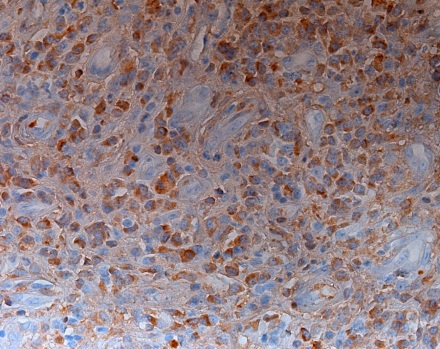 | 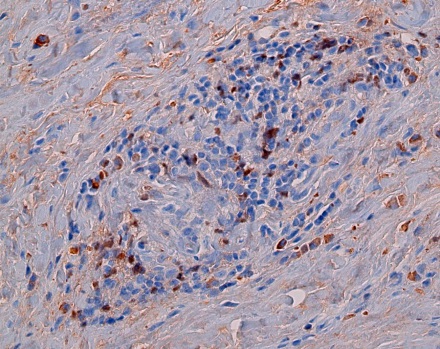 | 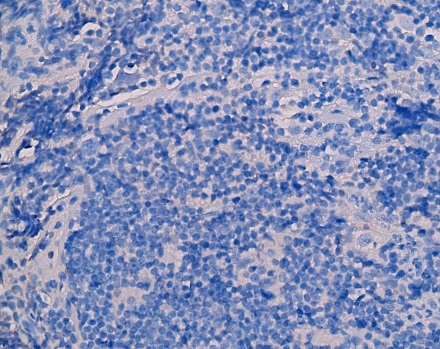 |
| **IgG4** | 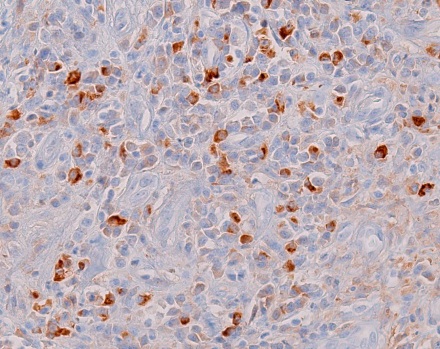 | 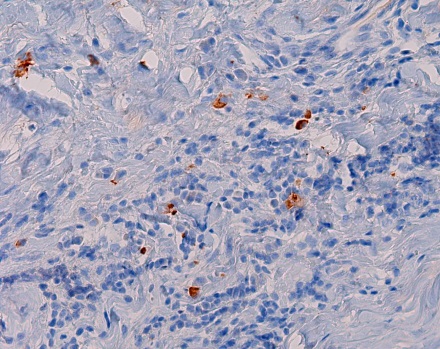 | 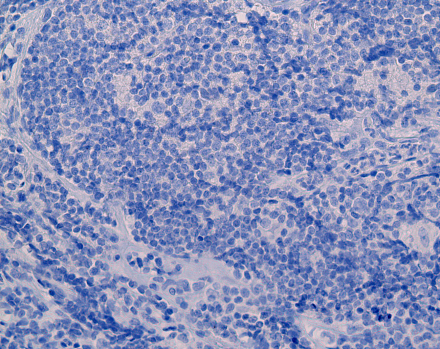 |

| **Table S1.** Comparison of gene expression in IgG4+ and IgG4- orbit adipose tissues. Listed are probe sets with at least a1.5-fold difference and also a false discovery rate adjusted p-value of <0.05 | | | | |
| --- | --- | --- | --- | --- |
| **Probe Set ID** | **Gene Symbol** | **Gene Title** | **Fold Change** | **FDR p value** |
| ***Probe sets with increased levels in IgG4+ tissue*** | | | | |
| 237625_s_at | --- | --- | 4.08 | 0.023 |
| 224589_at | XIST | X inactive specific transcript (non-protein coding) | 3.86 | 0.032 |
| 211639_x_at | IGH; IGHA1; IGHA2; IGHD; IGHG1; IGHG3; IGHG4; IGHM; IGHV4-31 | immunoglobulin heavy locus; immunoglobulin heavy constant alpha 1; immunoglobulin heavy constant alpha 2 (A2m marker); immunoglobulin heavy constant delta; immunoglobulin heavy constant gamma 1 (G1m marker); immunoglobulin heavy constant gamma 3 (G3m marker); immunoglobulin heavy constant gamma 4 (G4m marker); immunoglobulin heavy constant mu; immunoglobulin heavy variable 4-31 | 3.33 | 0.029 |
| 216829_at | IGK; IGKC | immunoglobulin kappa locus; immunoglobulin kappa constant | 3.06 | 0.013 |
| 205242_at | CXCL13 | chemokine (C-X-C motif) ligand 13 | 2.99 | 0.045 |
| 242020_s_at | ZBP1 | Z-DNA binding protein 1 | 2.80 | 0.048 |
| 234477_at | IGHA1; IGHV4-31 | immunoglobulin heavy constant alpha 1; immunoglobulin heavy variable 4-31 | 2.52 | 0.022 |
| 226811_at | FAM46C | family with sequence similarity 46, member C | 2.52 | 0.031 |
| 206478_at | KIAA0125 | KIAA0125 | 2.51 | 0.038 |
| 224404_s_at | FCRL5 | Fc receptor-like 5 | 2.44 | 0.041 |
| 216211_at | --- | --- | 2.43 | 0.012 |
| 205884_at | ITGA4 | integrin, alpha 4 (antigen CD49D, alpha 4 subunit of VLA-4 receptor) | 2.40 | 0.049 |
| 217227_x_at | IGLV1-44 | immunoglobulin lambda variable 1-44 | 2.40 | 0.039 |
| 216541_x_at | IGHG1; IGHM | immunoglobulin heavy constant gamma 1 (G1m marker); immunoglobulin heavy constant mu | 2.18 | 0.045 |
| 223565_at | MZB1 | marginal zone B and B1 cell-specific protein | 2.13 | 0.011 |
| 211908_x_at | IGK | immunoglobulin kappa locus | 2.11 | 0.040 |
| 204562_at | IRF4 | interferon regulatory factor 4 | 2.08 | 0.026 |
| 1558561_at | HM13 | histocompatibility (minor) 13 | 2.01 | 0.002 |
| 211648_at | IGHG1; IGHM | immunoglobulin heavy constant gamma 1 (G1m marker); immunoglobulin heavy constant mu | 1.98 | 0.034 |
| 208083_s_at | ITGB6 | integrin, beta 6 | 1.96 | 0.043 |
| 221159_at | --- | --- | 1.96 | 0.026 |
| 240854_x_at | --- | --- | 1.92 | 0.027 |
| 201688_s_at | TPD52 | tumor protein D52 | 1.86 | 0.019 |
| 225435_at | SSR1 | signal sequence receptor, alpha | 1.86 | 0.020 |
| 1558519_at | RPE | ribulose-5-phosphate-3-epimerase | 1.85 | 0.011 |
| 1556643_at | LOC100507535 | uncharacterized LOC100507535 | 1.85 | 0.035 |
| 1555351_s_at | PPHLN1 | periphilin 1 | 1.82 | 0.021 |
| 1557436_at | XKR6 | XK, Kell blood group complex subunit-related family, member 6 | 1.82 | 0.033 |
| 206925_at | ST8SIA4 | ST8 alpha-N-acetyl-neuraminide alpha-2,8-sialyltransferase 4 | 1.82 | 0.029 |
| 235567_at | RORA | RAR-related orphan receptor A | 1.80 | 0.046 |
| 1553670_at | INTS4 | integrator complex subunit 4 | 1.80 | 0.009 |
| 1555846_a_at | --- | --- | 1.80 | 0.028 |
| 1558710_at | --- | --- | 1.80 | 0.045 |
| 216573_at | IGLV1-44 | immunoglobulin lambda variable 1-44 | 1.79 | 0.040 |
| 239629_at | --- | --- | 1.79 | 0.027 |
| 201689_s_at | TPD52 | tumor protein D52 | 1.79 | 0.048 |
| 1566032_at | --- | --- | 1.78 | 0.029 |
| 229419_at | FBXW7 | F-box and WD repeat domain containing 7, E3 ubiquitin protein ligase | 1.77 | 0.030 |
| 224430_s_at | MTO1 | mitochondrial tRNA translation optimization 1 | 1.77 | 0.030 |
| 214984_at | LOC100271836; LOC101060596; SLC7A5P1 | smg-1 homolog, phosphatidylinositol 3-kinase-related kinase (C. elegans) pseudogene; serine/threonine-protein kinase SMG1-like; solute carrier family 7 (amino acid transporter light chain, L system), member 5 pseudogene 1 | 1.76 | 0.014 |
| 227539_at | GNA13 | guanine nucleotide binding protein (G protein), alpha 13 | 1.76 | 0.035 |
| 201197_at | AMD1 | adenosylmethionine decarboxylase 1 | 1.76 | 0.011 |
| 240118_at | --- | --- | 1.75 | 0.045 |
| 230392_at | --- | --- | 1.74 | 0.034 |
| 1562612_at | --- | --- | 1.74 | 0.019 |
| 242918_at | NASP | nuclear autoantigenic sperm protein (histone-binding) | 1.74 | 0.025 |
| 206854_s_at | MAP3K7 | mitogen-activated protein kinase kinase kinase 7 | 1.74 | 0.042 |
| 201741_x_at | SRSF1 | serine/arginine-rich splicing factor 1 | 1.73 | 0.020 |
| 1554478_a_at | HEATR3 | HEAT repeat containing 3 | 1.73 | 0.038 |
| 1556183_at | ANKRD36BP2 | ankyrin repeat domain 36B pseudogene 2 | 1.73 | 0.045 |
| 224520_s_at | BEST3 | bestrophin 3 | 1.73 | 0.037 |
| 239757_at | ZFAND6 | zinc finger, AN1-type domain 6 | 1.73 | 0.029 |
| 1555762_s_at | RBM15 | RNA binding motif protein 15 | 1.72 | 0.025 |
| 215470_at | GTF2H2B | general transcription factor IIH, polypeptide 2B (pseudogene) | 1.71 | 0.048 |
| 242723_at | OTTHUMG00000183913; RP11-932O9.10 | NULL; NULL | 1.71 | 0.027 |
| 1556657_at | --- | --- | 1.70 | 0.031 |
| 201287_s_at | SDC1 | syndecan 1 | 1.70 | 0.041 |
| 241240_at | --- | --- | 1.69 | 0.013 |
| 237803_x_at | --- | --- | 1.68 | 0.006 |
| 217540_at | NXPE3 | neurexophilin and PC-esterase domain family, member 3 | 1.68 | 0.022 |
| 215314_at | --- | --- | 1.68 | 0.034 |
| 1557036_at | ZBTB1 | zinc finger and BTB domain containing 1 | 1.67 | 0.016 |
| 206316_s_at | KNTC1 | kinetochore associated 1 | 1.67 | 0.041 |
| 244250_at | ANXA6 | annexin A6 | 1.66 | 0.004 |
| 239798_at | --- | --- | 1.65 | 0.033 |
| 217384_x_at | IGHV3-53; IGHV3-53; IGHV3-66; IGHV3-66; IGHV3-72; IGHV3-72 | NULL; immunoglobulin heavy variable 3-53; immunoglobulin heavy variable 3-66; NULL; immunoglobulin heavy variable 3-72; NULL | 1.64 | 0.043 |
| 205816_at | ITGB8 | integrin, beta 8 | 1.64 | 0.029 |
| 226444_at | --- | --- | 1.63 | 0.036 |
| 1558105_a_at | SLC9A7 | solute carrier family 9, subfamily A (NHE7, cation proton antiporter 7), member 7 | 1.63 | 0.035 |
| 220104_at | ZC3HAV1 | zinc finger CCCH-type, antiviral 1 | 1.62 | 0.040 |
| 215565_at | AC104699.1; OTTHUMG00000155125 | NULL; NULL | 1.62 | 0.032 |
| 213743_at | CCNT2 | cyclin T2 | 1.62 | 0.036 |
| 1562255_at | SYTL3 | synaptotagmin-like 3 | 1.62 | 0.039 |
| 228810_at | CCNYL1 | cyclin Y-like 1 | 1.61 | 0.036 |
| 240964_at | --- | --- | 1.59 | 0.020 |
| 244610_x_at | --- | --- | 1.59 | 0.030 |
| 221286_s_at | MZB1 | marginal zone B and B1 cell-specific protein | 1.59 | 0.025 |
| 209412_at | TRAPPC10 | trafficking protein particle complex 10 | 1.57 | 0.035 |
| 224406_s_at | FCRL5 | Fc receptor-like 5 | 1.57 | 0.036 |
| 51228_at | RBM12B | RNA binding motif protein 12B | 1.56 | 0.020 |
| 222473_s_at | ERBB2IP | erbb2 interacting protein | 1.56 | 0.029 |
| 1555350_at | PPHLN1 | periphilin 1 | 1.56 | 0.016 |
| 215992_s_at | RAPGEF2 | Rap guanine nucleotide exchange factor (GEF) 2 | 1.54 | 0.014 |
| 230724_s_at | C11orf57 | chromosome 11 open reading frame 57 | 1.54 | 0.042 |
| 202979_s_at | CREBZF | CREB/ATF bZIP transcription factor | 1.54 | 0.036 |
| 204207_s_at | RNGTT | RNA guanylyltransferase and 5'-phosphatase | 1.53 | 0.042 |
| 207100_s_at | VAMP1 | vesicle-associated membrane protein 1 (synaptobrevin 1) | 1.53 | 0.045 |
| 1557504_at | --- | --- | 1.53 | 0.045 |
| 1562349_at | TRAPPC2 | trafficking protein particle complex 2 | 1.53 | 0.028 |
| 243432_at | CHL1-AS2 | CHL1 antisense RNA 2 | 1.52 | 0.024 |
| 207600_at | KCNC3 | potassium voltage-gated channel, Shaw-related subfamily, member 3 | 1.52 | 0.043 |
| 241041_at | --- | --- | 1.52 | 0.045 |
| 1557966_x_at | MTERFD2 | MTERF domain containing 2 | 1.52 | 0.014 |
| 204444_at | KIF11 | kinesin family member 11 | 1.52 | 0.050 |
| 1569325_at | ARPC5 | actin related protein 2/3 complex, subunit 5, 16kDa | 1.52 | 0.046 |
| 241995_at | DGUOK | deoxyguanosine kinase | 1.52 | 0.022 |
| 1563023_at | --- | --- | 1.52 | 0.040 |
| 236202_at | --- | --- | 1.51 | 0.030 |
| 204872_at | TLE4 | transducin-like enhancer of split 4 (E(sp1) homolog, Drosophila) | 1.51 | 0.044 |
| 1570352_at | ATM | ataxia telangiectasia mutated | 1.51 | 0.013 |
| 242384_at | --- | --- | 1.51 | 0.022 |
| 229795_at | --- | --- | 1.51 | 0.031 |
| 208200_at | IL1A | interleukin 1, alpha | 1.51 | 0.019 |
| 228367_at | ALPK2 | alpha-kinase 2 | 1.51 | 0.031 |
| 204435_at | NUPL1 | nucleoporin like 1 | 1.50 | 0.046 |
| 1557131_at | SSSCA1-AS1 | SSSCA1 antisense RNA 1 (head to head) | 1.50 | 0.011 |
| ***Probe sets with decreased levels in IgG4+ tissue*** | | | | |
| 227299_at | CCNI | cyclin I | -1.50 | 0.011 |
| 210848_at | --- | --- | -1.51 | 0.029 |
| 205379_at | CBR3 | carbonyl reductase 3 | -1.51 | 0.040 |
| 216963_s_at | GAP43 | growth associated protein 43 | -1.52 | 0.014 |
| 238855_at | AHNAK | AHNAK nucleoprotein | -1.52 | 0.025 |
| 220894_x_at | PRDM12 | PR domain containing 12 | -1.52 | 0.048 |
| 1558502_s_at | DNM3 | dynamin 3 | -1.53 | 0.030 |
| 1566784_at | NSF | N-ethylmaleimide-sensitive factor | -1.53 | 0.049 |
| 227376_at | GLI3 | GLI family zinc finger 3 | -1.54 | 0.024 |
| 232085_at | MAPK8IP3 | mitogen-activated protein kinase 8 interacting protein 3 | -1.55 | 0.037 |
| 218692_at | SYBU | syntabulin (syntaxin-interacting) | -1.56 | 0.033 |
| 222013_x_at | FAM86A | family with sequence similarity 86, member A | -1.56 | 0.018 |
| 226645_at | KLF2 | Kruppel-like factor 2 (lung) | -1.56 | 0.033 |
| 226417_at | RHOB | ras homolog family member B | -1.57 | 0.013 |
| 230341_x_at | ADAMTS10 | ADAM metallopeptidase with thrombospondin type 1 motif, 10 | -1.59 | 0.029 |
| 222899_at | ITGA11 | integrin, alpha 11 | -1.59 | 0.029 |
| 206128_at | ADRA2C | adrenoceptor alpha 2C | -1.60 | 0.043 |
| 203792_x_at | PCGF2 | polycomb group ring finger 2 | -1.61 | 0.036 |
| 233812_at | LINC00028 | long intergenic non-protein coding RNA 28 | -1.61 | 0.026 |
| 1555380_at | ADAMTS4 | ADAM metallopeptidase with thrombospondin type 1 motif, 4 | -1.62 | 0.013 |
| 229207_x_at | RNF187 | ring finger protein 187 | -1.62 | 0.017 |
| 216904_at | COL6A1 | collagen, type VI, alpha 1 | -1.64 | 0.029 |
| 208333_at | LHX5 | LIM homeobox 5 | -1.65 | 0.025 |
| 1553572_a_at | CYGB | cytoglobin | -1.67 | 0.010 |
| 206869_at | CHAD | chondroadherin | -1.68 | 0.027 |
| 227404_s_at | EGR1 | early growth response 1 | -1.71 | 0.044 |
| 226765_at | SPTBN1 | spectrin, beta, non-erythrocytic 1 | -1.75 | 0.047 |
| 226578_s_at | DUSP1 | dual specificity phosphatase 1 | -1.75 | 0.014 |
| 1559960_x_at | SYCE1L | synaptonemal complex central element protein 1-like | -1.77 | 0.030 |
| 1567679_at | SNORA74A | small nucleolar RNA, H/ACA box 74A | -1.77 | 0.013 |
| 207008_at | CXCR2 | chemokine (C-X-C motif) receptor 2 | -1.82 | 0.038 |
| 241090_at | OTTHUMG00000177008; RP11-304L19.4 | NULL; NULL | -1.90 | 0.004 |
| 1567277_at | CTTN | cortactin | -2.06 | 0.025 |
| 210226_at | NR4A1 | nuclear receptor subfamily 4, group A, member 1 | -2.27 | 0.035 |
| 201044_x_at | DUSP1 | dual specificity phosphatase 1 | -2.34 | 0.038 |
| 217187_at | MUC5AC | mucin 5AC, oligomeric mucus/gel-forming | -2.77 | 0.015 |

| **Table S2.** Comparison of gene expression in IgG4+ and IgG4- lacrimal gland tissues. Listed are probe sets with at least a1.5-fold difference and also a false discovery rate adjusted p-value of <0.05 | | | | |
| --- | --- | --- | --- | --- |
| **Probe Set ID** | **Gene Symbol** | **Gene Title** | **Fold Change** | **FDR p value** |
| ***Probe sets with increased levels in IgG4+ tissue*** | | | | |
| 228599_at | MS4A1 | membrane-spanning 4-domains, subfamily A, member 1 | 3.51 | 0.004 |
| 221969_at | PAX5 | paired box 5 | 3.48 | 0.005 |
| 217422_s_at | CD22 | CD22 molecule | 2.87 | 0.016 |
| 1552280_at | TIMD4 | T-cell immunoglobulin and mucin domain containing 4 | 2.83 | 0.009 |
| 219014_at | PLAC8 | placenta-specific 8 | 2.83 | 0.030 |
| 209995_s_at | TCL1A | T-cell leukemia/lymphoma 1A | 2.74 | 0.031 |
| 231093_at | FCRL3 | Fc receptor-like 3 | 2.67 | 0.026 |
| 243781_at | --- | --- | 2.63 | 0.015 |
| 1553196_a_at | FCRL3 | Fc receptor-like 3 | 2.56 | 0.043 |
| 1558662_s_at | BANK1 | B-cell scaffold protein with ankyrin repeats 1 | 2.55 | 0.012 |
| 38521_at | CD22 | CD22 molecule | 2.48 | 0.028 |
| 222915_s_at | BANK1 | B-cell scaffold protein with ankyrin repeats 1 | 2.44 | 0.020 |
| 1553369_at | FAM129C | family with sequence similarity 129, member C | 2.39 | 0.040 |
| 221601_s_at | FAIM3 | Fas apoptotic inhibitory molecule 3 | 2.37 | 0.023 |
| 235400_at | FCRLA | Fc receptor-like A | 2.37 | 0.017 |
| 1564310_a_at | PARP15 | poly (ADP-ribose) polymerase family, member 15 | 2.35 | 0.047 |
| 205544_s_at | CR2 | complement component (3d/Epstein Barr virus) receptor 2 | 2.27 | 0.032 |
| 35974_at | LRMP | lymphoid-restricted membrane protein | 2.26 | 0.009 |
| 1558185_at | CLLU1; LOC100507616 | chronic lymphocytic leukemia up-regulated 1; uncharacterized LOC100507616 | 2.20 | 0.005 |
| 243468_at | PATE1 | prostate and testis expressed 1 | 2.19 | 0.036 |
| 204674_at | LRMP | lymphoid-restricted membrane protein | 2.19 | 0.008 |
| 211861_x_at | CD28 | CD28 molecule | 2.18 | 0.038 |
| 204581_at | CD22 | CD22 molecule | 2.14 | 0.009 |
| 219517_at | ELL3 | elongation factor RNA polymerase II-like 3 | 2.10 | 0.011 |
| 220999_s_at | CYFIP2 | cytoplasmic FMR1 interacting protein 2 | 2.09 | 0.032 |
| 219667_s_at | BANK1 | B-cell scaffold protein with ankyrin repeats 1 | 2.07 | 0.009 |
| 215925_s_at | CD72 | CD72 molecule | 2.05 | 0.030 |
| 214369_s_at | RASGRP2 | RAS guanyl releasing protein 2 (calcium and DAG-regulated) | 2.05 | 0.041 |
| 205671_s_at | HLA-DOB | major histocompatibility complex, class II, DO beta | 2.04 | 0.041 |
| 210279_at | GPR18 | G protein-coupled receptor 18 | 2.01 | 0.022 |
| 206760_s_at | FCER2 | Fc fragment of IgE, low affinity II, receptor for (CD23) | 1.99 | 0.034 |
| 223750_s_at | TLR10 | toll-like receptor 10 | 1.98 | 0.022 |
| 224406_s_at | FCRL5 | Fc receptor-like 5 | 1.98 | 0.003 |
| 230983_at | FAM129C | family with sequence similarity 129, member C | 1.95 | 0.023 |
| 228343_at | POU2F2 | POU class 2 homeobox 2 | 1.94 | 0.017 |
| 206478_at | KIAA0125 | KIAA0125 | 1.93 | 0.007 |
| 228426_at | CLEC2D | C-type lectin domain family 2, member D | 1.93 | 0.031 |
| 227030_at | IKZF3 | IKAROS family zinc finger 3 (Aiolos) | 1.92 | 0.020 |
| 1565034_s_at | AFF3 | AF4/FMR2 family, member 3 | 1.92 | 0.035 |
| 1552634_a_at | ZNF101 | zinc finger protein 101 | 1.92 | 0.048 |
| 221239_s_at | FCRL2 | Fc receptor-like 2 | 1.90 | 0.048 |
| 1559688_at | GRAPL | GRB2-related adaptor protein-like | 1.89 | 0.049 |
| 1569481_s_at | SNX22 | sorting nexin 22 | 1.88 | 0.029 |
| 1552892_at | TNFRSF13C | tumor necrosis factor receptor superfamily, member 13C | 1.84 | 0.017 |
| 1560524_at | GRAPL | GRB2-related adaptor protein-like | 1.84 | 0.034 |
| 208206_s_at | RASGRP2 | RAS guanyl releasing protein 2 (calcium and DAG-regulated) | 1.82 | 0.019 |
| 205367_at | SH2B2 | SH2B adaptor protein 2 | 1.82 | 0.011 |
| 224193_s_at | FCRL2 | Fc receptor-like 2 | 1.81 | 0.030 |
| 235661_at | POU2F2 | POU class 2 homeobox 2 | 1.81 | 0.009 |
| 235777_at | ANKRD44 | ankyrin repeat domain 44 | 1.80 | 0.036 |
| 208496_x_at | HIST1H3A; HIST1H3B; HIST1H3C; HIST1H3D; HIST1H3E; HIST1H3F; HIST1H3G; HIST1H3H; HIST1H3I; HIST1H3J | histone cluster 1, H3a; histone cluster 1, H3b; histone cluster 1, H3c; histone cluster 1, H3d; histone cluster 1, H3e; histone cluster 1, H3f; histone cluster 1, H3g; histone cluster 1, H3h; histone cluster 1, H3i; histone cluster 1, H3j | 1.80 | 0.049 |
| 219471_at | KIAA0226L | KIAA0226-like | 1.80 | 0.020 |
| 213638_at | PHACTR1 | phosphatase and actin regulator 1 | 1.79 | 0.012 |
| 210448_s_at | P2RX5 | purinergic receptor P2X, ligand-gated ion channel, 5 | 1.78 | 0.043 |
| 232112_at | RALGPS2 | Ral GEF with PH domain and SH3 binding motif 2 | 1.78 | 0.007 |
| 228518_at | IGHG1; IGHM | immunoglobulin heavy constant gamma 1 (G1m marker); immunoglobulin heavy constant mu | 1.77 | 0.012 |
| 206296_x_at | MAP4K1 | mitogen-activated protein kinase kinase kinase kinase 1 | 1.76 | 0.028 |
| 242458_at | RALGPS2 | Ral GEF with PH domain and SH3 binding motif 2 | 1.75 | 0.018 |
| 204777_s_at | MAL | mal, T-cell differentiation protein | 1.74 | 0.042 |
| 207777_s_at | SP140 | SP140 nuclear body protein | 1.74 | 0.016 |
| 205735_s_at | AFF3 | AF4/FMR2 family, member 3 | 1.74 | 0.036 |
| 214554_at | HIST1H2AG; HIST1H2AH; HIST1H2AI; HIST1H2AK; HIST1H2AL; HIST1H2AM | histone cluster 1, H2ag; histone cluster 1, H2ah; histone cluster 1, H2ai; histone cluster 1, H2ak; histone cluster 1, H2al; histone cluster 1, H2am | 1.74 | 0.013 |
| 236395_at | --- | --- | 1.72 | 0.037 |
| 239122_at | --- | --- | 1.72 | 0.008 |
| 219112_at | FNIP1; RAPGEF6 | folliculin interacting protein 1; Rap guanine nucleotide exchange factor (GEF) 6 | 1.72 | 0.044 |
| 238063_at | TMEM154 | transmembrane protein 154 | 1.72 | 0.018 |
| 236191_at | --- | --- | 1.70 | 0.020 |
| 209199_s_at | MEF2C | myocyte enhancer factor 2C | 1.70 | 0.048 |
| 227189_at | CPNE5 | copine V | 1.69 | 0.018 |
| 227198_at | AFF3 | AF4/FMR2 family, member 3 | 1.68 | 0.022 |
| 1557051_s_at | HOTAIRM1 | HOXA transcript antisense RNA, myeloid-specific 1 | 1.67 | 0.035 |
| 232286_at | --- | --- | 1.66 | 0.032 |
| 230128_at | CKAP2 | cytoskeleton associated protein 2 | 1.66 | 0.047 |
| 232140_at | LOC100132352 | FSHD region gene 1 pseudogene | 1.66 | 0.028 |
| 220918_at | RUNX1-IT1 | RUNX1 intronic transcript 1 (non-protein coding) | 1.66 | 0.020 |
| 239074_at | GRAPL | GRB2-related adaptor protein-like | 1.65 | 0.019 |
| 232034_at | LINC00537 | long intergenic non-protein coding RNA 537 | 1.65 | 0.039 |
| 244172_at | --- | --- | 1.64 | 0.007 |
| 218949_s_at | QRSL1 | glutaminyl-tRNA synthase (glutamine-hydrolyzing)-like 1 | 1.63 | 0.012 |
| 209828_s_at | IL16 | interleukin 16 | 1.63 | 0.037 |
| 235523_at | CTC1 | CTS telomere maintenance complex component 1 | 1.62 | 0.025 |
| 227607_at | STAMBPL1 | STAM binding protein-like 1 | 1.61 | 0.043 |
| 235372_at | FCRLA | Fc receptor-like A | 1.61 | 0.032 |
| 221690_s_at | NLRP2 | NLR family, pyrin domain containing 2 | 1.59 | 0.040 |
| 205642_at | CNTRL | centriolin | 1.59 | 0.046 |
| 239214_at | LOC100130458 | uncharacterized LOC100130458 | 1.58 | 0.040 |
| 241278_at | --- | --- | 1.58 | 0.045 |
| 240718_at | --- | --- | 1.57 | 0.011 |
| 1554108_at | --- | --- | 1.56 | 0.019 |
| 227749_at | POU2F2 | POU class 2 homeobox 2 | 1.54 | 0.025 |
| 210347_s_at | BCL11A | B-cell CLL/lymphoma 11A (zinc finger protein) | 1.54 | 0.039 |
| 235401_s_at | FCRLA | Fc receptor-like A | 1.53 | 0.022 |
| 239244_at | LOC100507616 | uncharacterized LOC100507616 | 1.53 | 0.028 |
| 231656_x_at | OSBPL10 | oxysterol binding protein-like 10 | 1.52 | 0.015 |
| 214562_at | HIST1H4A; HIST1H4B; HIST1H4C; HIST1H4D; HIST1H4E; HIST1H4F; HIST1H4H; HIST1H4I; HIST1H4J; HIST1H4K; HIST1H4L; HIST2H4A; HIST2H4B; HIST4H4 | histone cluster 1, H4a; histone cluster 1, H4b; histone cluster 1, H4c; histone cluster 1, H4d; histone cluster 1, H4e; histone cluster 1, H4f; histone cluster 1, H4h; histone cluster 1, H4i; histone cluster 1, H4j; histone cluster 1, H4k; histone cluster 1, H4l; histone cluster 2, H4a; histone cluster 2, H4b; histone cluster 4, H4 | 1.52 | 0.041 |
| 216734_s_at | CXCR5 | chemokine (C-X-C motif) receptor 5 | 1.51 | 0.041 |
| 222307_at | PDCD4-AS1 | PDCD4 antisense RNA 1 | 1.51 | 0.043 |
| 225021_at | ZNF532 | zinc finger protein 532 | 1.50 | 0.002 |
| ***Probe sets with decreased levels in IgG4+ tissue*** | | | | |
| 212793_at | DAAM2 | dishevelled associated activator of morphogenesis 2 | -1.52 | 0.033 |
| 202068_s_at | LDLR | low density lipoprotein receptor | -1.53 | 0.028 |
| 201148_s_at | TIMP3 | TIMP metallopeptidase inhibitor 3 | -1.57 | 0.034 |
| 221747_at | TNS1 | tensin 1 | -1.60 | 0.014 |
| 36829_at | PER1 | period circadian clock 1 | -1.61 | 0.009 |
| 214110_s_at | LOC654342 | lymphocyte-specific protein 1 pseudogene | -1.67 | 0.009 |
| 218245_at | TSKU | tsukushi, small leucine rich proteoglycan | -1.68 | 0.014 |
| 229238_at | C17orf97 | chromosome 17 open reading frame 97 | -1.83 | 0.011 |
